# Diabetic Socks: A Systematic Review of the Literature and Commercially Available Products

**DOI:** 10.1002/dmrr.70138

**Published:** 2026-02-21

**Authors:** Prabhuraj D. Venkatraman, Giorgio Orlando, Peter R. Culmer, Rory P. Turnbull, Katherine Bradbury, Irantzu Yoldi, Jenny Corser, David A. Russell, Andrew J. M. Boulton, Neil D. Reeves

**Affiliations:** ^1^ Manchester Fashion Institute Faculty of Arts and Humanities Manchester Metropolitan University Manchester UK; ^2^ Department of Sport and Exercise Sciences, Institute of Sport, Faculty of Science and Engineering Manchester Metropolitan University Manchester UK; ^3^ School of Mechanical Engineering University of Leeds Leeds UK; ^4^ School of Psychology University of Southampton Southampton UK; ^5^ Department of Allied and Public Health, School of Health Sport and Bioscience University of East London London UK; ^6^ Leeds Institute of Clinical Trials Research Faculty of Medicine and Health University of Leeds Leeds UK; ^7^ Diabetes Endocrinology and Metabolism Centre Manchester Royal Infirmary Manchester UK; ^8^ Diabetes Research Institute University of Miami Miami Florida USA; ^9^ Medical School Faculty of Health and Medicine Lancaster University Lancaster UK

**Keywords:** diabetic foot ulcer, diabetic neuropathy, footwear, knitted fabric and diabetic socks

## Abstract

**Aims:**

In recent years, various diabetic socks have been developed and introduced to help prevent diabetes related foot ulcers (DFUs). However, the question of which type of sock is most suitable for individuals with diabetes has not been adequately addressed. This review systematically explores the current evidence regarding the performance and structural properties of diabetic socks, along with a narrative analysis of commercial socks.

**Methods:**

Four databases, including Web of Science, PubMed/Medline, CINAHL (EBSCO), and Google Scholar, were examined, resulting in the selection of nine articles. Methodological quality of the clinical trials was assessed using the Downs and Black tool. Additionally, 17 commercially available socks were selected for analysis.

**Results:**

Among the selected articles, four were clinical trials, two mixed‐design studies, and three laboratory‐based investigations. The clinical studies exhibited poor quality and were unable to demonstrate significant outcomes. Laboratory‐based studies indicated that functional finishes on yarns could prevent microbial growth and that the blend of fibres and the structure of the socks significantly affect the overall performance. Commercially available socks reporting new types of fibres lack clinical evidence to support their claims, complicating the understanding of their performance in people with diabetes.

**Conclusions:**

Current evidence regarding the structure and function of diabetic socks is limited, with clinical trials characterised by poor methodological quality. Technical suggestions for diabetic sock design are based on limited evidence from laboratory‐based studies, highlighting gaps in the literature. This review highlights the need for further investigation to establish the evidence base for diabetic socks in supporting diabetic foot care.

## 1 Introduction

The International Diabetes Federation (IDF) has estimated that out of the 537 million people living with diabetes worldwide, 19%–34% will develop a diabetic foot ulcer (DFU) at some point in their lives [1]. Foot ulceration is the leading cause of lower‐limb amputations globally, severely impacting the quality of life and life expectancy of people with diabetes [1]. Approximately 20% of individuals with a DFU will require either minor or major lower‐extremity amputations, with five‐year mortality rates of 46% and 56%, respectively. Recurrence rates for DFUs are alarmingly high, with 40% recurring within 1 year and 65% within 3 years [1]. These outcomes are further exacerbated in the presence of chronic kidney disease [2]. The management of DFUs and their complications requires comprehensive, multidisciplinary, and long‐term care, making it not only a significant healthcare challenge but also a major financial burden due to the high costs of treatment [1]. It is estimated that NHS England allocates £1 billion annually to treating DFUs and their sequelae [3]. The International Working Group on the Diabetic Foot (IWGDF) recommends the use of therapeutic footwear for the prevention and treatment of DFU [4]. Indeed, consistent use of therapeutic footwear has been shown to significantly reduce plantar pressures and prevent DFU recurrence in people with diabetes [5]. While considerable attention has been given to the benefits of therapeutic footwear, the most suitable socks for patients with diabetes and how they should be recommended based on the DFU risk are still insufficiently discussed and understood, with only a systematic review exploring the role of socks in damping plantar pressure [6]. Nevertheless, diabetic socks may offer multiple benefits for individuals at risk of DFUs, by reducing vertical foot pressure (proof of concept for this has already been shown in people with DFU [7]), by potentially lowering the risk of infections via antibacterial yarns, and by promoting thermoregulation of the skin and blood flow through non‐restrictive, seamless welts. However, these benefits still need further investigation in patients with DFUs. The effectiveness of these socks may depend on several complex factors, including the sock's structure, materials, and design features. Key considerations include the type of fibres used (e.g., natural, synthetic, or biopolymer), yarn construction (e.g., single, double, textured, wrapped, or elastic), functional finishes (e.g., antimicrobial or anti‐odour treatments), knitted structures (e.g., single jersey, ribbed, or terry), and the overall design to ensure a proper fit for the feet and legs (e.g., gradient shaping).

In recent years, many specialised socks, known as ‘diabetic socks,’ have been developed and marketed for individuals with diabetes. These socks may feature an optimal elastic fit, thermal comfort, seamless construction, and enhanced breathability. However, despite the wide availability of these socks, their clinical effectiveness in preventing DFU and managing the complications of the foot in diabetes has yet to be assessed. There is no common understanding of which combination of design features (e.g., fibre type, yarns) is present or required in a diabetic sock, nor whether these appear consistently across the literature and commercial products. The absence of a comprehensive overview of the materials and structural variations of these socks complicates efforts to guide people with diabetes in selecting the most appropriate options for their needs.

This systematic review and narrative synthesis of the scientific literature aims to explore the structure, performance, and properties of socks for individuals with diabetes. It will comprehensively evaluate the structural features, fibre and yarn blends, various knitting techniques, and finishes applied to diabetic socks to synthesise the available evidence for optimal sock designs for people with diabetes. In addition to a systematic review of the scientific literature, a detailed overview of the structure and performance of current commercially available diabetic socks will be undertaken.

## 2 Materials and Methods

Following a search of the most relevant databases in textile science and the PROSPERO register, we found no previously published systematic reviews on our topic of interest. As the review aimed to explore the properties and materials of socks developed for people with diabetes, it did not meet the health‐related outcome requirement for PROSPERO registration.

### 2.1 Search Strategy

**FIGURE 1:**
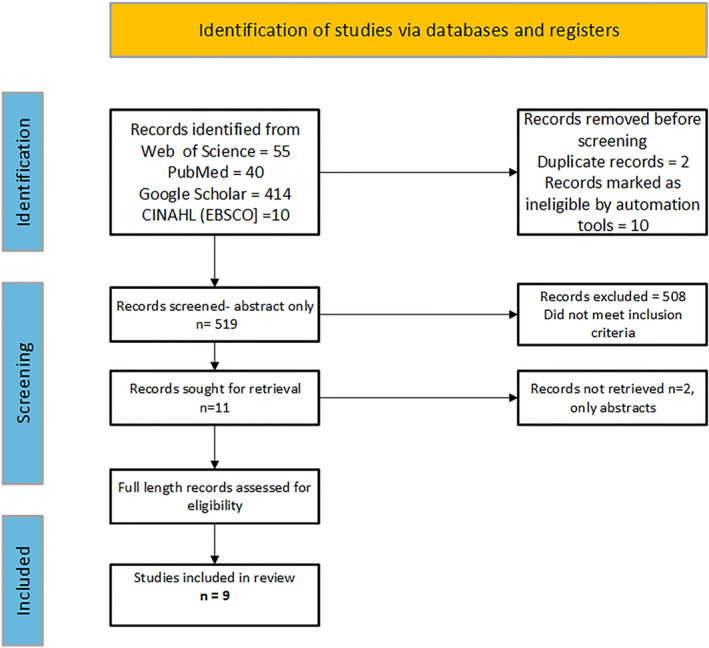
Flow diagram of the article selection process.

This systematic review adhered to the Preferred Reporting Items for Systematic Reviews and Meta‐Analyses (PRISMA) guidelines [7] (Figure 1). Three experienced researchers (PV, GO, and NR) conducted the systematic review and independently identified, selected, and reviewed research articles, and extracted data. From 2000 to 2024, four databases‐ Web of Science, PubMed/Medline, CINAHL (EBSCO), and Google Scholar‐were thoroughly examined to identify the articles based on the selection criteria. The reference lists of the included studies were also examined to identify other potentially relevant works. Reviewers used Rayyan Software to select, screen, and exclude duplicate articles and were blinded during the selection of articles. The following search terms were used: ‘diabetic socks' OR 'socks' AND ‘diabetes mellitus' OR ‘type 1 diabetes’ OR ‘type 2 diabetes’ OR ‘diabetic neuropathy' OR ‘diabetic foot ulcer. In addition to the review of the scientific literature, in this research, we provide a comprehensive and systematic evaluation of the design features (including fibre composition, yarn type and knit structures) of 17 commercially available socks designed and marketed specifically for people with diabetes. Similar to the eligibility criteria for a systematic review of the scientific literature, only socks without any embedded technologies were examined.

### 2.2 Study Selection: Inclusion and Exclusion Criteria

The inclusion and exclusion criteria for this systematic review are provided in Table 1. Briefly, the review included studies on socks termed or considered ‘diabetic socks,’ or those specifically developed for individuals with diabetes, with primary outcomes addressing textile structure, performance, and properties (including comfort), as well as the types of yarns used in the socks. Commentaries, review articles and case studies were not considered. The main exclusion criteria included any sensing technology embedded in the socks (i.e., measuring pressure and temperature), research published in languages other than English, and socks specifically designed for treating active DFU or developed for treating oedema were also excluded. Due to the specific terminology used in the selected articles, a glossary of terms is provided in the Supporting Information S1 [8, 9, 10].

**TABLE 1:** Study's selection criteria.

Inclusion criteria	Exclusion criteria
1. Socks termed or considered as ‘diabetic socks', or socks specifically developed for people with diabetes	1. Use of sensing technology (e.g., measuring temperature, pressure, humidity) 2. Sock garment used as part of the treatment for people with active DFU 3. Socks focussed on reducing oedema 4. Socks not aimed specifically for people with diabetes 5. Does not allow a shoe to be worn 6. Studies exclusively focussing on antibacterial and infection prevention 7. Qualitative research—case study
2. Type: Original research, pilot or feasibility studies or perspective articles.	
3. Reports on material aspects of socks:	
a) textile structure	
b) textile performance and properties (including reports on functional parameters),	
c) type of yarns used,	
d) textile material,	
e) Worn in combination with a shoe	
f) Has closed‐toe area	
g) Worn by people with diabetes generally, including those with type 1, type 2	
4. Qualitative and quantitative studies	
5. Written in English	
6. Published between 2000 and 2024	

### 2.3 Data Extraction

Reviewers (PV, GO and NR) were not blinded to the journal title or authors, and information was recorded and extracted using a structured Microsoft Excel file. This process involved documenting the origin and publication year of each study, as well as demographic information, including sample size, gender, and age. Clinical details, including the type of diabetes and presence of long‐term complications, were also recorded. Additionally, the reviewers captured outcomes, along with the quantitative and qualitative tools utilised, the primary findings, and the conclusions of each study.

### 2.4 Quality Assessment

The methodological quality of the clinical studies (*n* = 6) was assessed using the Downs & Black tool developed for randomised and non‐randomised clinical trials [11]. Two reviewers (PV and GO) were responsible for the quality check. The Downs & Black tool is characterised by high internal consistency, test‐retest and inter‐rater reliability. This tool utilises a scoring system comprising 27 questions to assess the study quality (10 items), external and internal validity (3 items), bias (7 items), confounding and selection bias (6 items) and the power of the study (1 item). The score obtained from each study was divided by 32 (the highest score) and multiplied by 100 to provide a percentage. The following cut‐off values were adopted: high (66.7% or higher), fair (between 50.0% and 66.6%) and low (< 50.0%), as reported in previous reviews [12, 13]. Discrepancies were resolved through discussion, and in cases of differing judgements, a third author (NR) was consulted for resolution (Table 2).

**TABLE 2:** Quality assessment of clinical studies using a tool developed by Downs and Black [11].

Study	Is the aim of the study clear?	Are the main outcomes clearly described?	Are the characteristics of patients clearly described	Are the interventions of interest clearly described?	Are the confounders clearly described?	Are the main findings of the study clearly described?	Have adverse events been reported?	Have the characteristics of patients lost to follow‐up been described?	Were actual probability values reported in the analysis?	Did the study recruit a representative of the entire population?	Did the study report on those subjects who were prepared to participate?
Tarbuk et al. [14]	0	1	0	1	0	1	0	0	0	0	U
Soltanzadeh et al. [15]	1	1	1	1	0	1	0	U	1	0	U
Garrow et al. [16]	1	1	1	1	U	1	U	U	1	0	1
Van Amber et al. [17]	1	1	1	1	0 (U)	1	0	0	1	0 (U)	1
Soh et al. [18]	1	1	1	1	0 (U)	1	0	0	1	0 (U)	0 (U)
Cüreklibatır Encan et al. [19]	1	1	1	1	0 (U)	1	0	0	1	0 (U)	0 (U)

Study	Were the analyses adjusted for different lengths of follow‐up	Did the study use appropriate statistical tests?	Were there compliance with interventions reliable?	Were the main outcome measures accurate and reliable	Were cases or controls recruited from the same population	Were cases or controls recruited over the same time	Were the subjects randomised to intervention groups	Was randomised intervention concealed from subjects and clinicians?	Was the analysis adjusted to follow‐up subjects?	Does the study have sufficient statistical power to detect clinically relevant effects?	Were the main outcome measures used accurately?	Total score based on 32 items (%)
Tarbuk et al. [14]	0	0	0	0	0	0	0	0	0	0	U	3 (9.3%)
Soltanzadeh et al. [15]	0	1	1	1	0	0	0	0	U	0	1	10 (31.2%)
Garrow et al. [16]	U	1	1	1	0	0	1	U	U	0	1	11 (34.3%)
Van Amber et al. [17]	1	1	1	1	1	1	1	0	0 (U)	0	1	15 (46.9%)
Soh et al. [18]	0 (U)	0 (U)	0	1	0 (U)	0 (U)	0	0	0 (U)	0	1	8 (25%)
Cüreklibatır Encan et al. [19]	0 (U)	1	0	1	0 (U)	0 (U)	0 (U)	0 (U)	0	0	0 (U)	8 (25%)

*Note:* Assessment key: Yes–1; No–0; Unable to determine: 0 (U). The following questions were not presented in the table, as they do not apply to the studies reviewed and scored ‘0’ under quality assessment. (1) Does the study provide estimates of the random variability in the data for the main outcomes? (2) Were the staff, places, and facilities where the patients were treated representative of the treatment the majority of patients receive? (3) In trials and cohort studies, do the analyses adjust for different lengths of follow‐up of patients, or in case‐control studies, is the time period between the intervention and outcome the same for cases and controls? (4) If any of the results of the study were based on ‘data dredging’, was this made clear? (5) Were the patients in different intervention groups (trials and cohort studies) or were the cases and controls (case‐control studies) recruited from the same population?

### 2.5 Commercial Diabetic Socks

In addition to the scientific literature, a review of the current commercially available (March–July 2024) diabetic socks was undertaken. The inclusion and exclusion criteria, where applicable, were the same as those described above for the systematic review of the scientific literature.

## 3 Results

### 3.1 Summary of Systematic Literature Search and Quality Assessment

The literature search identified 520 articles, of which only nine were eligible for inclusion (Figure 1). The main characteristics of the nine studies are presented in Table 3. The publication year of these studies ranged from 2000 to 2024. Of the selected research articles, two employed a mixed design characterised by both laboratory and clinical studies [14, 17], four were exclusively clinical in nature [15, 16, 18, 19] and the remaining three consisted solely of laboratory studies [20, 21, 22]. Five studies involved participants with diabetes, either uncomplicated diabetes or those at high risk of DFU [14, 16, 17, 18, 19] and one involved healthy participants [15]. This latter study was included in the systematic review since it met the inclusion criteria of socks termed or considered as ‘diabetic socks’.

**TABLE 3:** Overview of research on socks designed for diabetic patients.

Authors	Aims	Methodology	Results	Conclusions
Tarbuk et al. [14]	To test durability comfort and the ability to reduce antimicrobial infection among six socks developed with and without modified cotton yarn.	Mixed study design including laboratory study and clinical study. Lab study: Durability tested pre‐ and post‐15 wash cycles; Antimicrobial protection of the socks tested against gram‐positive *Staphylococcus aureus* and Gram‐negative *Klebsiella pneumoniae* and against microfungus *Candida albicans*; Objective measurements of friction using the fabric surface tester: Friction coefficient measured. Clinical study: 10 people with diabetes, including type 1 diabetes (*n* = 3), type 2 diabetes (*n* = 4), and gestational diabetes (*n* = 3), were recruited. Parameters evaluated over 10 h of wear time: Comfort; Liquid adsorbency. Socks were made from: 100% cotton; Cotton/minerals—embedded with crushed volcanic minerals; Cotton/cocona ‐ infused with activated carbon made from coconut shells; Cotton/tribomechanical activated zeolite; Single knit structure—summer; Double‐knit winter socks.	Lab study: Unmodified cotton socks: Not effective on Gram‐positive and Gram‐negative bacteria or micro‐fungus before and after 15 wash cycles; Modified cotton yarn with natural zeolite, natural minerals, and active carbon (summer and winter socks): Effective on gram‐positive bacteria before and after 15 wash cycles; Samples of cotton blend with Cocona and modified yarns with natural minerals (winter socks only): Effective on Gram‐negative bacteria before and, to some extent, after 15 wash cycles; Socks made from modified cotton: Effective on micro fungus both before and after 15 wash cycles; Socks made with mineral/zeolite/active carbon particles: Improved absorbency Friction: Similar across all sock types. Clinical study: Cotton socks: Odour after 10 h; Cotton socks modified with minerals: No sweating or odour after 10 h; Socks modified with active carbon: Same properties after 15 wash cycles; Socks with natural minerals: Lower absorbency leading to sweating and odour after 15 washes.	Cotton socks modified with natural minerals and activated carbon increased absorbency, reducing sweating and mitigating odour. This effect created a dry environment, which prevented the development of bacteria and fungi.
Darwish et al. [20]	To describe and compare seven commercial diabetic socks.	Laboratory study of different types of commercially available diabetic socks and comparison between the foot and leg sections of the socks. Analysis: Sock material composition; Yarn type; Air permeability; Strength; Abrasion resistance; Thermal resistance; Water vapour permeability.	Comparison among socks: 6/7 socks were composed of cellulose fibres (5 from cotton and one from bamboo); 5/7 socks contained > 90% cotton; 4/7 contained polyester fibres; 3/7 contained nylon; 7/7 contained Lycra (range: 1%–30%). Comparison of leg and foot sections: 5/7 socks produced from yarns of different constructions for the foot and leg sections; The yarn count was always finer in the foot section than in the leg section. The foot section was produced from a single yarn or single cover Lycra; Double cover Lycra is one of the main yarns for the leg part, with two, three or four covered yarns added in varying proportions; 7/7 socks, the number of wales per inch and the number of courses per inch were greater in the foot compared to the leg section.	Four materials were used in commercial diabetic socks in varying proportions: Cellulose fibres (cotton or bamboo), polyester, nylon and Lycra. The yarn types and number of yarns vary across the foot and leg sections of the socks. Finer yarns are used on the foot section of the socks. It is proposed that to optimise sock properties and, therefore, comfort, diabetic sock use should be focused separately on summer and winter use.
Van Amber et al. [17]	Investigate whether socks manufactured from different fibre types and fabric structures contributed to changes in skin health indicators in people with diabetes.	Mixed study design including laboratory study and clinical study. Lab study: Comparison between two experimental socks and a control sock (commercially available). Analysis: Mass per unit area; Moisture regain; Fabric thickness; Cyclic compression. Clinical study: 20 people with type 1 and type 2 diabetes at high risk of foot ulceration were recruited. Participants first wore the control socks for 4 weeks, then wore the single Jersey socks (Group 1) for 18 weeks. Finally, Group 2 (*n* = 19) wore the terry socks for 18 weeks, while Group 3 wore control socks. Skin health indicators: Skin temperature; Transepidermal water loss; Stratum corneum hydration; Skin hardness. Two experimental socks were composed of 90% merino wool and 10% nylon (polyamide). The socks were identical except for the structure under the heel and in the toe box: in one sock, this was a single Jersey, and in the other, a terry pile. A commercial control sock was selected for comparison, which was marketed for people with diabetes and recommended by diabetes Australia.	Lab study: Terry socks: Greater thickness, mass per unit area and moisture regain compared to the single Jersey and control socks; Single Jersey socks: Thinner than control socks; Mass per unit area: similar between single Jersey and control socks; Moisture regain: Higher in single Jersey compared to control socks. Clinical study: Skin temperature: Highest when wearing the terry socks and lowest wearing the single Jersey socks; There was no effect of sock type on transepidermal water loss, stratum corneum hydration or skin hardness; The greatest reduction in sock thickness after wearing: Terry (−10%) followed by the single Jersey (−7%) sock.	Terry socks are thicker, with greater mass and regained more moisture compared to the other tested socks. In wearer trials, sock type had little effect on the parameters measured except skin temperature, which was higher with the terry socks.
Soh et al. [18]	Investigate the pressure‐reducing effects of the StepEase diabetic socks in people with diabetes and evaluate their satisfaction.	Clinical study including 32 diabetic patients at high‐risk for diabetic foot ulceration, prospective, followed up over 12 weeks. Plantar pressure measurements: Baseline, 6 and 12 weeks of wearing socks with and without wearing the StepEase. Plantar pressure was assessed using the Novel Pedar‐X system both barefoot and wearing the StepEase diabetic socks. Participant's usage and satisfaction with the socks were assessed using a questionnaire. StepEase socks were made from microspheres of EVA (Ethylene Vinyl Acetate) of 500–1000 μm packed together in a padding which was enclosed by a fabric material (cotton Lycra). Microspheres disperse within the pouch allowing for pressure to be distributed evenly. StepEase socks appeared more like foot coverings rather than socks that could be worn along with shoes.	Foot pressure: Baseline measure: The StepEase socks significantly reduced peak foot pressure compared to the barefoot condition at 7 out of 8 foot sites. Pressure reductions ranged between 53% and 117%. After 6 and 12 weeks of wearing: The peak foot pressure reductions observed at baseline were maintained, with all foot sites except the left toe displaying significantly lower peak pressures with the StepEase socks compared to barefoot. Comfort: ‘Very satisfied’: 13% of participants ‘Satisfied’: 65% ‘Neutral’: 10% ‘Unsatisfied’: 13%	Wearing padded socks (StepEase) around the home reduced peak foot pressures and this pressure reduction was maintained for 12 weeks of follow‐up. 78% of participants were either satisfied or very satisfied with the socks. These socks were designed to be worn around the home only and would not be compatible with wearing within most footwear. In this sense they might be regarded as indoor foot coverings rather than conventional socks to be worn within footwear.
Cüreklibatır Encan et al. [21]	To develop and test seven different types of sock samples to determine the optimal diabetic sock.	*Laboratory study* Thermal conductivity; Air permeability; Water vapour permeability; Coefficient of friction; Abrasion resistance; Recovery after compression; Seven different sock samples: 95% cotton/5% synthetic yarn; 90% cotton/10% polyamide; 80% cotton/20% lyocell; 100% polyester; 50% viscose/50% acrylic; 35% acrylic/35% polyester/30% viscose; 100% acrylic. The above seven sock types were all produced with Jersey and piquet fabric structures.	*Thermal conductivity*: Highest (i.e., heat transfer) in the sock with 95% cotton/5% synthetic yarn and lowest with 100% acrylic, with no difference between Jersey and piquet structures. *Air permeability* (i.e., airflow through the fabric): Highest in the sock with 100% polyester and lowest in the socks with i) 50% viscose/50% acrylic and ii) 100% acrylic. Socks with the piquet fabric structure had a higher air permeability compared to those with the Jersey structure. *Water vapour permeability* (i.e., movement of water vapour through the sock): Highest in the sock with 100% polyester and lowest in the sock with 100% acrylic. Socks with a Jersey structure had a higher water vapour permeability compared to those with a piquet structure. *Coefficient of friction* (i.e., resistance between the sock and the skin): Lowest in the socks with 80% cotton/20% lyocell and highest in the socks with 100% acrylic. Lowest coefficient of friction for socks with a Jersey structure compared to those with piquet structure. Abrasion resistance (i.e., ability to resist wear over lifespan of the socks): Highest in socks with 90% cotton/10% polyamide and lowest in socks with 50% viscose/50% acrylic. Socks with a Jersey structure had a higher abrasion resistance compared to those with piquet structure. Recovery after compression: Higher in socks with i) 50% viscose/50% acrylic, ii) 100% polyester, iii) 100% acrylic all made with Jersey structure and iv) 100% acrylic made with piquet structure. Socks with a Jersey structure had higher recovery after compression than those with a piquet structure.	Socks made from 100% polyester yarn were determined to be the most suitable for diabetic use due to their ability to prevent overheating, reduce sweating, protect the skin, and offer increased durability and overall protection.
Cüreklibatır Encan and Marmarali [22]	The study aimed to develop and test different types of socks to determine the optimal material and knit structure suitability for a diabetic sock.	*Laboratory study* 9 different socks were tested: Fabric thickness, Thermal resistance and conductivity, Air permeability, Water vapour permeability, Coefficient of friction, Abrasion resistance, Recovery after compression Longitudinal elasticity. Socks were developed using three different types of yarn (all Ne 30/1) cotton, acrylic and polyester and three different fabric structures Jersey, piquet and terry. The above combination of yarn type and knit structure yielded 9 different types of socks being developed for laboratory testing. An elastic yarn (70 den nylon/20 den elastane) was also fed to the needles beside the ground yarns in all samples.	*Thermal resistance* (i.e., capacity to retain heat within the sock): Knit structure was the most important, being highest in the socks made with a terry knit structure compared to those with piquet or Jersey structures. Polyester and acrylic yarns showed higher thermal resistance compared to cotton yarns for each of the three knit structures. Thermal conductivity: Yarn type more important than knit structure, with cotton yarns showing the highest thermal conductivity (greatest ability to transfer heat) compared to polyester and acrylic. Thermal conductivity was similar across all three yarn structures (Jersey, piquet and terry). *Air permeability* (i.e., passage of air through): Highest in socks with the piquet structure and lowest in those with the terry knitted structure. Polyester yarn showed the highest air permeability compared to cotton and acrylic yarns. Water vapour permeability: Jersey knit showed the highest water vapour permeability, and terry structure showed the lowest water vapour permeability. There was no difference between the three yarn fibre types. Coefficient of friction: The Jersey structure showed the lowest coefficient of friction, while the terry structure showed the highest. The type of yarn did not influence the coefficient of friction. Abrasion resistance: Highest in socks with a terry‐knitted structure when using polyester yarns. Recovery after compression: Highest with the Jersey structure and lowest with the terry yarn structure. Yarn structure was determined to be the most critical factor for recovery after compression. Longitudinal elasticity: Yarn structure was the most important factor, with socks made using the Jersey structure showing the highest longitudinal elasticity and the terry structure the lowest.	The optimal diabetic socks should be made from polyester yarns using piquet knit for the leg and foot, with Jersey structure for the heel and foot due to its lower friction, vapour permeability, and high recovery from compression.
Cüreklibatır Encan et al. [19]	This study investigated the effects of a diabetic sock on the prevention of diabetic foot ulcers within a 6‐month prospective study.	Clinical study conducted in 11 participants with diabetes, including individuals both with and without a prior history of foot ulceration. *Measures*: Foot pressure measurements: weeks 1, 4, 12 and 24 with participants both barefoot and wearing the socks; Patients were monitored for signs of diabetic foot ulceration. Participants' perceptions of comfort: Baseline and after the 6‐month follow‐up period. The sock was produced using a combination of single Jersey and piquet structure. A single Jersey was used on the sole, and a piquet structure was used on the upper foot and leg. A polyester yarn (Ne 70/1) was fed during knitting and finished with antibacterial and antifungal properties.	Foot pressure *Plantar pressure*: decreased over the follow‐up period while wearing the socks at the fourth and fifth metatarsal head. The statistical significance of the overall sample was not clear. No patients developed a diabetic foot ulcer over the 6‐month follow‐up period. *Patient's perceptions* of satisfaction with the socks changed positively over the 6‐month wear period. *Patient's opinions* about thermal absorptivity and surface roughness either remained constant or changed negatively over the 6‐month wear period. Patients believed that the socks diminished the lack of feeling in their feet, reduced itching, did not provide a warmer feeling to the feet and did not like the white colour of the socks.	Newly developed socks reduced the pressure at the fourth and fifth metatarsals in diabetes patients over a 6‐month follow‐up period. Patients' satisfaction with the socks generally changed positively over the 6‐month wear period.
Soltanzadeh et al. [15]	To test the effectiveness of seven developed socks for reducing plantar foot pressure while walking.	Clinical study on 10 healthy participants (male *n* = 5). Seven different sock types were worn in a randomised order, and the barefoot condition was used as a control. The Gaitview AFA‐50 system was used to measure plantar pressures while people walked at their self‐selected speed. Seven different socks were developed. The structures of the toe, heel, foot and leg were plain knitted fabric. The same cotton (20/1 Ne) and Lycra yarns were used for all socks. The sole of the socks varied for the seven different socks by using different fabric structures: i) Plain, ii) single cross‐tuck, iii) mock rib, iv) cross miss, v) mock rib, vi) double cross tuck and vii) double cross miss.	Foot pressure: All sock types reduced the mean plantar pressure compared to barefoot walking conditions, but this reached significance for only two sock types. Two sock types significantly reduced mean plantar foot pressure compared to barefoot walking in the toes and first metatarsal head region, these were the cross miss and mock rib socks.	All sock structures evenly re‐distributed the mean plantar pressure distribution compared to barefoot walking, but the cross miss knitted structure and mock rib knitted structure were the most effective. This was attributed to the fact that miss loops make the cross miss and mock rib structures like terry loops, which cause a cushioning and absorbent surface for the foot that helps to reduce plantar foot pressure.
Garrow et al. [16]	To investigate the efficacy of preventative foot care socks (PFC) for reducing plantar foot pressure in a group of patients with diabetes at high risk for foot ulceration.	Clinical study including 19 people with moderate to severe diabetic neuropathy. All patients were ulcer‐free at the time of participation. Foot pressure was assessed using the F‐scan in‐shoe system (Tekscan). Measurements were taken in participant's own footwear while walking along a flat 5‐m walkway. The order of testing either PFC or control (standard supermarket) socks was randomised before data collection. PFC socks had a double‐layer construction consisting of a padded outer layer to cushion the feet and a low‐friction fibre inner layer. PFC socks had a maximum thickness of 2.7 mm compared to 0.7 mm for the control socks. Standard ‘supermarket’ socks were used as a control.	Foot pressure: Mean plantar foot total pressure: Reduced by 9% in the PFC socks compared to control. In the forefoot area, plantar pressure reduced by 10% compared to control socks, attributed to a 14% increase in contact area with PFC socks.	PFC socks with a double layer were effective at reducing foot pressure across the whole foot and in the crucial forefoot region compared to a control (supermarket) sock. This reduction in foot pressure was primarily attributed to the increased contact area with the PFC sock.

Six clinical studies were assessed for quality among the nine articles. As shown in Table 4, the studies achieved an average score of 28.6% (SD = 12.4). These data indicate that clinical studies were characterised by a low level of quality.

**TABLE 4:** Commercially available diabetic socks.

S. No.	Diabetic socks type	Details reported by manufacturer/retailer
1	Cosy feet ‐ Coolmax socks	Ideal for large or swollen legs, fuller‐fitting knee‐high socks, extra room Coolmax seam‐free socks. The sock has a rib construction and is made of 87% cotton, 11% nylon and 2% Lycra [23]
2	Protect iT	Socks made from silver ions blended fibres offer antibacterial, antifungal, and anti‐odour properties. They also offer a comfortable grip with ventilating properties for effective thermal regulation [24]
3	Reflexa diabetic socks	Made from Celliant yarns that enhance oxygen levels, increase vascular flow, regulate body temperature and relieve pain. Sock promotes comfort using a non‐binding top and flat toe seam [25].
4	Skinnies UK therapeutic socks	Skinnies therapeutic Dermasock diabetic socks are made from viscose fibres, offering good 360‐degree stretch and comfort [26]
5	HJ diabetic socks	The socks are made from 80% wool fibres that offer insulating properties, are cool to wear in summer, and keep the feet warm during winter. They have a non‐elastic welt, terry knit structure for the feet, improved comfort in swollen ankles, and loose knit mesh to prevent restrictive blood flow. They are also recommended for oedema and sensitive feet. The Institute of Chiropodists and Podiatrists endorses them [27].
6	Orthofeet socks	Padded sole diabetic socks have a loose knit construction, super stretch, non‐constrictive, antibacterial, anti‐odour, seam‐free toe, excellent moisture‐wicking, and foot cushioning interior. They support blood circulation and are suitable for sensitive feet and feet with oedema. Fibre composition: 67% viscose rayon. 26% cotton, 6% polyester, 1% elastane [28].
7	Dr Segal's diabetic socks	The diabetic sock is cushioned (terry knit structure) and has a seamless toe closure. Its antimicrobial and moisture‐wicking properties help prevent infection and injury. Physicians design socks for diabetic and neuropathy patients for added stretch and comfort [29].
8	White EasyStretch Viasox diabetic socks	Socks with non‐binding technology can stretch up to 30 inches to ensure a perfect fit for all leg sizes. It has triple‐padded support for the sole/feet and promotes blood circulation. It comprises 44% polyester, 34% bamboo, 14% cotton and 8% Lycra [30].
9	Well‐healed	Socks are made from 30% cotton, 46% acrylic, 13% Coolmax, 2% Lycra and 9% polyester. It has a good stretch to accommodate different leg size, flat toe seam and is machine washable [31].
10	Thorlo socks	Offer a range of sportswear socks and provide diabetic socks with cushioned toes with seams, using Thorlon fibres that support moisture wicking and friction reduction. Socks are made from 83% Thorlon acrylic, 13% nylon and 4% elastic yarns [32].
11	Jobst socks	Socks made of acrylic yarn feel like cotton, with padding for the toe and heel [33].
12	TXG diabetic Cushion Socks	The sock has non‐binding top hand‐locked seams and is padded from heel to toe for comfort. It also has an open‐knitted structure for breathability. Made of 84% acrylic, 8.5% nylon and 7.5% Lycra [34].
13	LifeSock ‐ Difoprev	Difoprev system—the sock made from fibres with microcapsules containing moisturising agents ‐ *Pseudoalteromonas* ferment extract, a glycoprotein synthesised by the bacterium *Pseudoalteromonas antarctica*, NF3. Recommended for cutaneous treatment for patients suffering from diabetic neuropathy, it provides hydration and prevents dryness and hyperkeratosis of the foot/shin [35].
14	Silipos arthritis and diabetic gel socks	Socks provide optimum protection for neuropathic feet (nerve‐damaged feet caused by diabetes or arthritis). These cushioning socks help to reduce friction, abrasion, and shear forces while preventing ulceration and the formation of calluses. Silipos gel contains mineral oil that conforms to the body, offers a good fit and comfort, cushions, and moisturises the skin [36].
15	Truform Trusoft diabetic sock	Socks are made of acrylic fibre with a rib‐knit structure that offers comfort, stretch, and wicking properties. The fibres are finished with Aegis, which inhibits the growth of bacteria [37].
16.	Dr Scholl's diabetic socks	Dr Scholl's diabetes crew socks, made from polyester yarns, are comfortable, promote healthy circulation, and keep the feet dry, making them ideal for everyday wear. The socks have a smooth toe seam that prevents irritation and offers soft cushioning in the sole [38].
17	Podartis	Two different types of socks are offered for people with diabetes: ‘Energy Diab’ and ‘protection Diab’. Energy Diab uses Crabyon, a blend of viscose and chitosan with antibacterial and anti‐odour properties. The fibre composition is as follows: 39% polyester Coolmax, 25% cotton, 4% nylon, 13% PP Polypropylene—6% Viscose/Crabyon, 2% AG silver, and 1% EA Lycra. Claims include maintaining the right level of humidity and hydration and having haemostatic wound‐healing properties. Protection Diab, with its padded sole and foot, offers protection and shock absorbing effect, made from yarns finished with a bacteriostatic agent [39].

### 3.2 Literature Review Detailed Findings

A detailed description of the selected studies is presented in Table 3.

### 3.3 Mixed Designs

Tarbuk et al. [14] tested eight different types of socks (Table 3) developed with and without modified cotton yarn to understand their comfort level, the ability to reduce antimicrobial colonisation and liquid adsorbency (sweat, water surfactant). During lab analysis, cotton yarn modified during mercerisation with natural zeolite, natural minerals, and activated carbon (summer and winter socks) was reported to prevent the development of Gram‐positive bacteria on the sample both before and after 15 wash cycles. Similarly, cotton socks blended with Cocona (i.e., activated carbon yarn) and modified yarns with natural minerals (winter socks only) were reported to prevent the development of Gram‐negative bacteria before and to a certain extent after 15 wash cycles. In contrast, cotton socks (100%, without added minerals or activated carbon) did not prevent the growth of Gram‐positive or Gram‐negative bacteria or microfungus.

Tests to assess the liquid adsorption of the socks were performed in 10 people with diabetes of uncharacterised clinical status, who wore the socks for 10 h. Participants wore 100% cotton socks, which resulted in odour after 10 h, whereas cotton socks modified with minerals and activated carbon did not produce sweating or odour. After 15 wash cycles, socks modified with active carbon retained these properties, but those modified with natural minerals had lower absorbency, leading to sweating and odour.

Van Amber et al. [17] compared the fibre types (90% merino and 10% nylon) and fabric structure at the heel and toe (single jersey vs. terry pile) of two different experimental socks against a commercial control sock (84% cotton, 13% nylon and 3% elastane, made using terry knit structure) recommended by Diabetes Australia. In addition to lab‐based measures (e.g., mass per unit area, moisture regain), this study included an 18‐week user trial with people with diabetes at high risk of DFU to explore the impact of different socks on several skin health indicators (e.g., skin temperature and epidermal water loss). The terry socks had a significantly greater thickness, mass per unit area, and moisture regain than the single jersey and control socks. Skin temperature was highest when wearing the terry socks and lowest when wearing the single jersey socks. There was no effect of sock type on transepidermal water loss, stratum corneum hydration or skin hardness. After 18 weeks of wearing, the most significant reduction in sock thickness was observed in the terry (−10%), followed by the single jersey (−7%) sock. An important potential conflict of interest should be noted for Van Amber et al. [17] study since Wool Industry Research Ltd. funded this research. Furthermore, the presence and severity of autonomic neuropathy (people with autonomic neuropathy have reduced sweating ability) among the participants —which could have influenced the study outcomes and cannot be determined from the article.

### 3.4 Laboratory Studies

Three studies reported the outcomes of socks developed for individuals with diabetes [20, 21, 22]. Cüreklibatır et al. [21] developed and lab‐tested socks made from seven different yarn types, produced with both jersey and piquet fabric structures (all incorporating antibacterial properties with the active ingredients silver and zinc), resulting in 14 sock variations in total. It aimed to determine the optimal sock material for diabetic socks, considering their performance across various laboratory‐based material assessments. Thermal conductivity was highest (the greatest level of heat transfer) in the sock with 95% cotton/5% synthetic yarn, with no difference between jersey and piquet structures. Air and water vapour permeability were highest (highest airflow through the fabric) in the sock with 100% polyester (Table 3). Socks with a piquet fabric structure exhibited higher air permeability, whereas socks with a jersey structure showed higher water‐vapour permeability. For the coefficient of friction, the yarn structure rather than the yarn type was deemed most important, being the lowest (i.e., offering the least resistance between the sock and the skin) for socks with a jersey knitted structure compared to those with a piquet structure. The coefficient of friction was lowest in socks made with 80% cotton/20% lyocell, whereas abrasion resistance was highest (i.e., a greater ability to resist wear over the lifespan of the socks) in socks made with 90% cotton/10% polyamide. Socks with a jersey knitted structure had higher abrasion resistance (greater ability to resist wear over the lifespan of the socks) than those with a piquet structure. Recovery after compression was higher (better fabric resilience and recovery from compression, therefore better protection from high plantar pressures) in socks with i) 50% viscose/50% acrylic, ii) 100% polyester, iii) 100% acrylic, all made with jersey structure and iv) 100% acrylic made with piquet structure. Socks with a jersey knitted structure generally had higher recovery after compression than those with a piquet structure.

Much like the previous study, Cüreklibatır Encan and Marmarali [22] determined the optimal material and knit structure suitability for diabetic socks by analysing nine types of socks made from three different yarns (cotton, acrylic, and polyester) and three different knit structures (jersey, piquet, and terry). Sock‐knit structures play an essential role in determining thermal resistance (the capacity to retain heat within the sock), particularly terry knits with polyester and acrylic yarns, which exhibit high thermal resistance. The type of fibre used affected the thermal conductivity (ability to transfer heat) of the socks, with cotton yarns exhibiting higher conductivity than polyester and acrylic. Overall, fibre type had a greater effect on thermal conductivity than the sock knit structure. Yarn type also played a crucial role in air permeability (air movement through the fabric), where polyester yarns had higher permeability than cotton and acrylic. Similarly, water vapour permeability was higher in single jersey knits compared to terry knits. Sock‐knit structures affected the coefficient of friction, where terry knit offered higher resistance than single jersey knit. Terry knits with polyester yarns also showed high abrasion and low recovery after compression compared with single jerseys.

Darwish et al. [20] reported a comparative analysis of seven different types of commercial socks suitable for both summer and winter use, evaluating their structure, composition and properties. Five of the seven socks tested were produced from yarns of different constructions (Table 2) for the foot and leg sections. Fine yarns were noticed more in the foot section than in the leg sections. The fibre composition of the socks consisted of cotton, bamboo, polyester, nylon and Lycra. Lycra content varied from 1% to 6%, with some socks containing up to 30%, which is typically seen in elastic stockings. The mechanical properties, comfort, air permeability, and thermal properties varied between the foot and leg sections of the socks. The authors [20] noted that summer and winter socks should be designed separately to optimise various performance properties, such as air and vapour permeability, thermal transmission, fabric thickness, and comfort.

### 3.5 Clinical Studies

Soh et al. [18] investigated the pressure‐reducing effects of ‘StepEase’ diabetic socks (padding thickness of 1.25 cm) and patient satisfaction in 32 participants at high risk of DFU. The study reported that the mean peak plantar pressure was highest at the right forefoot and left heel, and there was a significant reduction in the mean peak pressure in the heel, midfoot, and metatarsal regions. The highest‐pressure reduction was observed in the right toe region, and the reduction in peak pressure remained significant at 6 weeks (−47%; −87.4 kPa) and 12 weeks (−49%; −91.9 kPa). Thirteen percent of the participants were ‘very satisfied’ with the sock, 65% were ‘satisfied’, 10% were ‘neutral’, and 13% were ‘unsatisfied’. Although the StepEase socks effectively reduced peak foot pressure, they were designed for indoor use only because of their size and thickness and are completely inappropriate for use with any footwear, representing a significant limitation.

Cüreklibatır Encan et al. [19] investigated the effects of a diabetic sock on foot pressure, DFU prevention and patient satisfaction within a 6‐month prospective study. They developed a unique sock made from 100% polyester yarn (76 denier), treated with silver ions to provide bacteriostatic and fungistatic effects. The sock featured a single jersey knit structure (1.05 mm thickness) in the sole and heel regions, and a piquet knit (1.28 mm thickness) in the upper foot and leg regions. Plantar pressure measurements were taken at four time points across 6 months, with measurements recorded in barefoot conditions with and without socks. The data show trends of decreasing plantar pressure while wearing the socks over the follow‐up periods at the fourth and fifth metatarsal heads; however, statistical significance could not be determined from this study. No patient developed a DFU over the study period. Participants reported that wearing the socks relieved itching and expressed a desire for the sock welt to be comfortable. Patients' satisfaction with the socks generally changed positively over the wear period.

Soltanzadeh et al. [15] developed and tested seven different types of socks to reduce foot pressure and foot ulcer risk in people with diabetes. All socks were developed with the toe, heel, foot, and leg regions made from plain knitted fabric using cotton (20/1 Ne) and Lycra (50 Nm) yarns, while the soles varied using different fabric structures (see Table 3). The mean plantar pressure decreased in all sock types during walking, but this reached significance for only two types in the toes and first metatarsal head region: the cross miss and mock rib socks. The cross‐miss knitted and mock rib knitted structures were deemed the most effective.

Garrow et al. [16] investigated the efficacy of preventive foot care socks (PFC) for reducing plantar foot pressure in patients with diabetes at high risk for foot ulceration. PFC socks had a maximum thickness of 2.7 mm compared to 0.7 mm for the control socks, which included a standard ‘supermarket’ sock. Foot pressure was assessed in participant's footwear while they walked along a flat 5‐m walkway. Total plantar foot pressure was reduced by 9% in the PFC socks compared with the control, which was largely attributed to an increased total foot contact area by 8% when wearing PFC socks. In the crucial forefoot area (the area of most DFUs), plantar pressure was reduced by 10% compared with control socks, attributed to a 14% increase in contact area with PFC socks.

## 4 Discussion

Three potential factors have been identified for diabetic socks that could influence their performance: (1) the selection of materials for sock production—optimum fibre and yarn blends; (2) the influence of yarns on sock‐knitted structures; and (3) the impact of functional finishes in preventing infections. However, evidence is weak, and studies are generally of poor quality.

### 4.1 Fibre and Yarn Blends

In sock design, an optimal fibre blend is essential for achieving the desired performance; therefore, different fibre types are preferred. For instance, adding cotton and polyester to Lycra provides comfort, durability, and stretch, and is a typical blend composition for socks. Lycra is an elastic filament that offers stretch and recovery properties, although no more than 8% is recommended for use. Recently, bamboo fibres have also been used in socks, as they are hypoallergenic, antibacterial, eco‐friendly, and act as a natural deodorising agent, while also being moisture absorbent [40] and often blended with cotton or nylon. The foot region of the socks requires greater elasticity, which explains why single‐ or double‐Lycra yarns are used. Additionally, double‐cover yarns are recommended for the leg region due to their greater elasticity [20, 21, 22, 41].

Wool fibres are protein fibres with low thermal conductivity and are hygroscopic, which will absorb and release moisture without feeling damp, and due to their natural fibre crimp, they trap air within their structure, which offers warmth and helps to regulate body temperature. Being highly hygroscopic, they feel drier to the touch than other fibres, such as polyester and cotton [42]. Due to these properties, they are preferred for winter months, specifically using merino wool, coarse wool, and acrylic fibres, which provide thermal and moisture transfer properties [8]. Fibre type, diameter, and packing density significantly affect a fabric's thermal resistance and liquid absorption capacity [43]. Acrylic fibres, also used in socks, have a lower mass than wool fibres, resulting in looser packing within the fabric structure and providing greater thermal and water vapour resistance. Coarse wool has a larger diameter (26 μm) than fine merino wool (19 μm) and acrylic fibres (19 μm), allowing it to retain more moisture between its interstitial spaces. Therefore, it is essential to balance the fibre blend ratio to optimise product performance (moisture transfer, thermal management and water vapour resistance) [43].

### 4.2 Influence of Yarns and Sock‐Knitted Structures

The type and fineness of yarns used in sock production impact the performance of socks and the knit structures formed, such as jersey, piquet, terry or rib knit structures. It also determines the thickness of the socks and various functional properties, including thermal conductivity, air permeability, water vapour permeability, coefficient of friction, abrasion resistance, and recovery after compression [44]. Jersey knits are typically thinner and exhibit high vapour permeability, moisture transfer, and thermal resistance properties. Similarly, piquet knits, which have a porous knit structure, also possess similar properties, such as moisture transfer, allowing free air flow through their interstitial spaces and exhibiting better air permeability compared to jersey knits [21].

**FIGURE 2:**
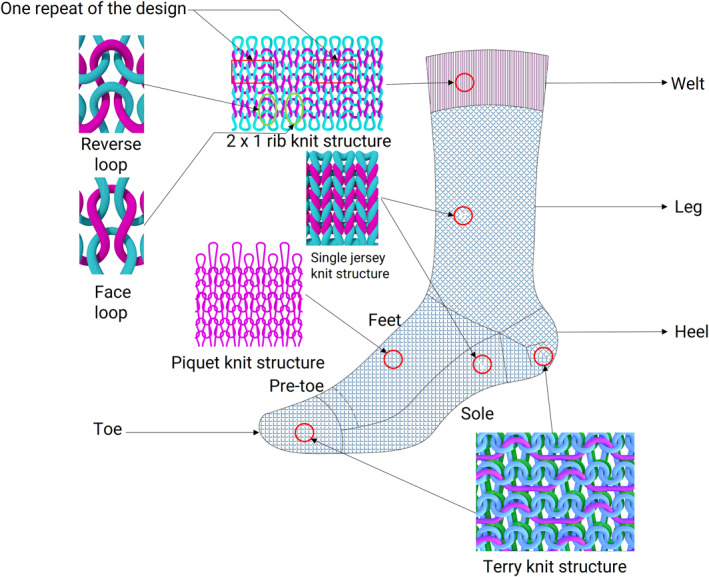
Technical suggestions for diabetic sock design.

It is worth noting that although yarns have different levels of fineness, knit structures, jersey, piquet, and terry exhibit distinct performance characteristics irrespective of the yarn types used (cotton, acrylic and polyester) [22]. Likewise, socks (of different structures such as jersey, piquet, and terry) made from polyester yarns have high air permeability and recovery after compression [22]. However, terry knit socks (made of cotton, acrylic and polyester yarns), due to their greater thickness, possess thermal resistance, abrasion resistance and low air and water permeability, making them more suitable for the sole region of the socks [21, 22, 42, 45]. Single jersey knit structures recover better after compression than piquet knits and are ideal for the foot zone in the sock [21] (Figure 2). Recovery from compression is a measure of fabric resilience and could protect the feet from plantar pressure [22, 41]. Compression‐to‐recovery of sock fabrics is affected by increased fabric density and thickness [46].

Socks made from merino wool fibres provide better cushioning due to superior recovery following compression. This affects the physical properties of the knit structures and provides energy‐absorbing properties [22, 41, 46], which could be an essential feature for reducing plantar pressure on the feet. Socks made from cross‐miss and mock rib structures also possess terry loops that are effective in reducing plantar pressure across all the toes and the first metatarsal head region; however, these findings have been reported only in healthy women [15, 47].

### 4.3 Effect of Functional Finishes on Sock Performance

Functional finishes refer to the treatment of fibres or yarns, which can affect the properties of socks and, in turn, their overall performance. There is only in vitro evidence indicating that socks made from polyester yarns finished with silver and zinc prevent bacterial growth [21] and antifungal activity [19].

Cotton yarns treated with minerals and blended with natural volcanic minerals may enhance the antibacterial and antifungal properties of winter socks, as observed in a lab study [14]. Cotton yarns finished with Cocona and cotton yarns modified with activated natural zeolites in micro‐ and nano‐particle forms demonstrated antibacterial and antifungal activity for winter socks and some antimicrobial resistance for summer socks. It is essential to note that winter socks with activated natural zeolites and summer socks with Cocona may lose their resistance to Gram‐negative bacteria (*K. pneumoniae*) after 15 washes [14]. This highlights that the finishes on the yarns used for sock production are less durable and require further clinical studies and development. These early in vitro studies [14, 20, 21] demonstrating the potential for antibacterial and antifungal efficacy need to be evaluated in patients with DFU.

## 5 Analysis of Commercially Available Socks for Diabetic Applications

A comprehensive analysis was conducted on the 17 commercially available socks specifically designed for individuals with diabetes to evaluate their performance. This study was undertaken to provide insights, in addition to the scientific literature, into the current offerings of ‘diabetic socks’ from commercial stakeholders and to identify prevailing trends in their design and development. Most (8/17) of them were made from cotton, viscose, nylon, and elastane fibres, including Cosyfeet, Viasox, Well‐healed, Jobst, Orthofeet, Skinnies, TXG diabetic and Dr Scholl's (Table 3). In contrast, several brands (7/17) reported using speciality yarns (ProtectiT, Reflexa, Dr Segal's socks, Lifesock Difoprev system, Silipos, Truform and Podartis). For example, Reflexa socks made unsubstantiated claims that they regulate temperature, promote vascular flow, and alleviate pain by incorporating Celliant yarns [30, 32, 35, 48]. Similarly, Coolmax yarns have been reported in some socks (Podartis) and claim to enhance moisture‐wicking properties using polyester filaments, which vary in fibre size and feature channels designed to transport moisture away from the skin [45, 47]. Only one company (HJ Diabetic socks) reported using wool fibres for additional thermal insulation and employed various knitted structures that were claimed to enhance comfort. These features are known to be particularly beneficial in providing warmth during the winter months, but alternatively may promote sweating and moisture development, contributing toward microbial growth during the summer months.

Several socks included padded structures or increased sole thickness, effectively raising the mass per unit area. Research has suggested that using merino wool fibres may reduce plantar pressure [43, 46, 48]. Ten brands emphasised the importance of a seamless toe region, as traditional seams can cause rubbing on the toes. Knit structures for commercial diabetic socks include terry pile knit for cushioning, open knit for breathability and rib knit for increased lateral stretch in the leg area. Most socks incorporate elastic filaments such as Lycra, with proportions typically ranging between 1% and 8%. For example, Viasox claims that its socks containing 8% Lycra can stretch up to 76.2 cm to fit various leg shapes [30]. While up to 8% Lycra is considered safe and effective, a higher proportion of elastic fibres (up to 30%) has also been reported [20]. Such a high level of compression may be considered excessive for everyday diabetic socks, with the potential for damage to fragile skin, due to an insensate foot.

Cotton, nylon, and elastic yarns have been used to enhance moisture‐wicking and reduce friction in socks, as seen with Thorlo padded socks [21, 32]. Additionally, functionally finished yarns may contain microcapsules for added moisturising and skin hydration and antibacterial and antifungal treatments to help prevent infections (LifeSock—Difoprev system) [35]. The majority of claims by the companies were unsupported by clinical evidence, highlighting the need for further work.

## 6 Literature Gaps and Technical Suggestions for Improving Sock Design

To our knowledge, this is the first systematic analysis of scientific literature, focussing specifically on the effectiveness of newly developed designs and materials for people at risk of DFU. Among the limited studies involving human participants, all exhibited poor methodological quality. This complicates efforts to draw conclusions regarding the efficacy of these socks. This review also revealed a notable gap in the literature regarding the recommended sock length (ankle, mid‐calf, or knee) and the optimal fibre and yarn composition for individuals with diabetes at risk of DFU. We also identified a complete absence of evidence addressing sock design for individuals with diabetes and a previous minor amputation, underscoring the need for future research.

Laboratory‐based research studies underscored the significance of fibre blend composition in sock construction, particularly the content of Lycra (ranging from 1% to 6%), as well as other fibre blends that include cotton, acrylic, polyester, and nylon [20, 21]. Notably, the finishing process of cotton with natural minerals and activated carbon has been shown to enhance resistance to bacterial growth [14]. Clinical studies are urgently needed to determine how these finishing processes influence infection prevention and management in individuals at risk of diabetic foot ulcers.

The knitted structure of the sock is a vital factor affecting overall performance; for example, variations in structure, such as single jersey, piquet, and terry, directly impact sock thickness. Thicker socks are known to retain moisture and maintain an optimal skin temperature [17]. Additionally, cross‐miss and mock rib knit structures, featuring terry‐pile loops, have been found to reduce plantar pressure across all toes [15]. Conversely, single jersey knit structures composed of polyester yarns provide a smoother surface, resulting in a lower coefficient of friction, as observed in a laboratory study [21].

The knit structure not only influences the thickness but also contributes to the comfort properties of the sock. Based on thickness, socks can be classified for seasonal applications, with thinner socks (< 1.3 mm) suited for summer and thicker options (> 2.25 mm) appropriate for winter [20]. It is also noted that socks may be designed with varying knit structures to impart specific functionalities across different zones, including the welt, leg, foot, and toes. As illustrated in Figure 2, the welt section should have a rib knit structure which offers considerable stretch and elasticity and holds the sock in the desired position of the leg, a single jersey knit for the leg zone, a piquet knit (has a porous knit structure) on the dorsal side of the feet, and a terry pile on the plantar side of the feet, which offers cushioning [22]. A seamless toe with a terry pile knit for improved comfort is recommended.

## Author Contributions

P.D.V., G.O., and N.D.R. conceived the review, conducted the systematic analysis, interpreted the data, and drafted the manuscript. A.J.M.B., P.R.C., R.P.T., K.B., I.Y., J.C., and P.R.C. interpreted the data and reviewed the manuscript. All authors have read and approved the final version of the manuscript. P.D.V., G.O., and N.D.R. are the guarantors of this work.

## Funding

The Engineering and Physical Sciences Research Council (EPSRC): EP/X001059/2 Digital Health: ‘Socksess' ‐ Smart Sensing Socks For Monitoring Diabetic Feet And Preventing Ulceration.

## Ethics Statement

The authors have nothing to report.

## Conflicts of Interest

The authors declare no conflicts of interest.

## Supporting information


Supporting Information S1

## Data Availability

Data sharing not applicable to this article as no datasets were generated or analysed during the current study.
