# Supplementary material for: Diabetic Socks: A Systematic Review of the Literature and Commercially Available Products
Source: Diabetes Metab Res Rev. 2026 Feb 21;42(2):e70138. doi: 10.1002/dmrr.70138 (PMC12924646; doi:10.1002/dmrr.70138)
Supplement: Supplementary file 1 — Supporting Information S1 [file DMRR-42-e70138-s001.docx]

**Diabetic Socks: A Systematic Review of the Literature and Commercially Available Products**

**Glossary of terms**

- **Adsorption:**  A process whereby the liquid is taken up between the fibre spaces on their surface rather than being held within the fibre structure
- **Back loop**: A knitted loop viewed such that it meshes through a previous loop away from the viewer.
- **Courses:**  Refers to horizontal rows of needle loops that the adjacent needles produce during the same knitting cycle.
- **Denier (den):** This is the unit of measure for measuring the linear density of fibres or yarn fineness. It is the mass in grams per 9000 metres of fibre length.
- **Diabetic socks**: Socks recommended for people with diabetes to reduce the risk of foot ulceration and support foot care.
- **English yarn count (Ne_C_):** This is an indirect yarn count system in which 840 yards weigh one pound (lb). For instance, if 10 x 840 yd hanks of cotton weigh 1lb, it is 10 Ne**_C_**. The higher the number, the finer the yarn.
- **Fibre:** Fibres are basic raw materials for a textile product which are from natural or synthetic sources, which have a defined length and diameter.
- **Face loo**p: It is a knitted loop viewed such that it meshes through a previous loop toward the viewer.
- **Knit:** It refers to a loop of yarn meshed at its base with a previously formed loop.
- **Knit structure**: A fabric made of yarn loops that are intermeshed to form a 3-dimensional structure.
- **Knitted fabric:** Has a knitted loop arranged in rows similar to woven fabric, termed courses or wales.
- **Knitting**: The process of forming a fabric by intermeshing a loop of yarn. There are two types of knitting – warp or weft knitting.
- **Miss knit:** It refers to a stitch that skipped the needle without forming a new loop, creating a gap in the fabric or patterns.
- **Mock rib knit:** It is a knit stitch pattern that offers a visual effect similar to a rib knit but with a simple construction. It is often achieved by knitting all stitches on the right side and alternating knit and purl stitches on the wrong side.
- **Moisture regain:** It is the ratio of mass of moisture in a textile to the oven-dry mass, usually expressed in percentage.
- **Plain knit or jersey:** It is produced by knitting needles in a single set, drawing the loops away from the technical back of the fabric and toward the technical face of a knit fabric.
- **Purl knit** is a knit structure in which the wales contain both the face and reverse meshed loops. The fabric is reversible and resembles the technical back of knit fabric.
- **Rib-knit:** This knit structure requires two sets of needles operating in parallel. Thus, wales of face stitches and wales of reverse stitches are knitted on each side of the fabric, making both sides similar or reversible.
- **Stitch**: It is a single loop of yarn created by the knitting process. A loop has three parts: two legs or sides and a crown part.
- **Stitch density:** It refers to the total number of loops in a measured area of fabric, usually the number of courses and wales.
- **Terry knit:** It is a knitted fabric made with an uncut looped pile, showing on the reverse side of some of the stitches. The plush loops are elongated sinker loops of the yarn at the back of the plated fabric.
- **Wales:** It refers to the vertical column of intermeshed needle loops produced by the same needle at successive knitting cycles.
- **Warp knitting:** It is a knitted fabric which creates vertical loops that intermesh in a zig-zag pattern, forming the loops in the vertical or lengthwise direction.
- **Weft knitting**: It is a knitted fabric with the loop of yarns moving horizontally, forming stitches across the width of the fabric.
- **Yarns**: A yarn is a twisted or interlocked strand of fibres made from synthetic filaments or natural staple fibres. These yarns are fundamental components of a fabric. They can be either knitted or woven to form the fabric.
